# FakeStack: Hierarchical Tri-BERT-CNN-LSTM stacked model for effective fake news detection

**DOI:** 10.1371/journal.pone.0294701

**Published:** 2023-12-01

**Authors:** Ashfia Jannat Keya, Hasibul Hossain Shajeeb, Md. Saifur Rahman, M. F. Mridha

**Affiliations:** 1 Department of Computer Science and Engineering, Bangladesh University of Business and Technology, Dhaka, Bangladesh; 2 Department of Computer Science & Engineering, American International University of Bangladesh, Dhaka, Bangladesh; University of Sindh, PAKISTAN

## Abstract

False news articles pose a serious challenge in today’s information landscape, impacting public opinion and decision-making. Efforts to counter this issue have led to research in deep learning and machine learning methods. However, a gap exists in effectively using contextual cues and skip connections within models, limiting the development of comprehensive detection systems that harness contextual information and vital data propagation. Thus, we propose a model of deep learning, FakeStack, in order to identify bogus news accurately. The model combines the power of pre-trained Bidirectional Encoder Representation of Transformers (BERT) embeddings with a deep Convolutional Neural Network (CNN) having skip convolution block and Long Short-Term Memory (LSTM). The model has been trained and tested on English fake news dataset, and various performance metrics were employed to assess its effectiveness. The results showcase the exceptional performance of FakeStack, achieving an accuracy of 99.74%, precision of 99.67%, recall of 99.80%, and F1-score of 99.74%. Our model’s performance was extended to two additional datasets. For the LIAR dataset, our accuracy reached 75.58%, while the WELFake dataset showcased an impressive accuracy of 98.25%. Comparative analysis with other baseline models, including CNN, BERT-CNN, and BERT-LSTM, further highlights the superiority of FakeStack, surpassing all models evaluated. This study underscores the potential of advanced techniques in combating the spread of false news and ensuring the dissemination of reliable information.

## Introduction

The phrase “fake news” typically denotes fabricated or misleading information that is presented as if it were legitimate news. The main purpose of it is to deceive or mislead readers, listeners, or viewers. It has become a major concern because of its ability to influence public opinion and decision-making processes. As online platforms for social interaction and the internet have grown in popularity, the task of differentiating real news from fake news has grown progressively challenging. As a result, increasing demand exists for reliable and precise ways to identify bogus news [1]. The rise of bogus news and misinformation turned into a serious problem for media professionals and policymakers, and it has created the need for automated techniques that can help identify and combat these issues [2]. One of the most critical reasons for the importance of automatic identification of bogus news is ensuring news sources’ integrity. Detecting bogus news is complicated because of the diverse nature of news articles, the speed at which they are circulated, and the limited resources available for verifying facts manually. Traditional methods of detecting false news focus on manual fact-checking or content analysis, which can be time-consuming and often lead to inaccuracies. Automated techniques have, therefore, become increasingly important for detecting fake news [3, 4].

Fake news detection has recently generated a lot of interest as a subject, and numerous approaches have been suggested in existing research literature [5]. The problem has been addressed extensively using machine learning and deep learning models [6]. Making use of deep learning-based approaches can help identify false information and stop it from spreading, leading to a more informed and knowledgeable society [7]. Improving the credibility of social media platforms is also essential. Social media platforms have been criticized for their role in the spread of fake news, and the use of deep learning-based approaches can help detect and prevent the spread of fake news, improving their credibility [8]. Finally, the use of deep learning-based approaches can reduce the manual efforts required for the identification and verification of news sources. This can up the effectiveness of news verification as well as lessen the workload on individuals and organizations [9].

Multiple research studies have demonstrated the efficacy of deep learning models in detecting fake news [10, 11]. Convolutional Neural Network (CNN) has proven to be effective in tasks involving text classification [12]. It has found application in diverse natural language processing tasks, among them the detection of fake news. CNNs excel in the realm of bogus news identification due to their capability to extract intricate features and patterns from textual data [13]. For instance, Liu et al. introduced a model based on CNNs to detect fake news, achieving an accuracy of 87.1% on the LIAR dataset [14]. Similarly, Zhang et al. presented a CNN-based model for bogus news identification, attaining an accuracy of 93.3% on the same data [12]. These outcomes underscore the efficacy of CNNs in detecting false news.

Long Short-Term Memory (LSTM) has also proven to be successful in tasks involving the modeling of sequences [15]. Roy et al. [16] introduced a hate speech detection model using LSTM and TF-IDF vectorization, demonstrating its superior accuracy in classifying hateful sentiments compared to various other models. Another study by Roy et al. [17] focused on combating rising phishing attacks by employing recurrent neural network models, including LSTM, Bidirectional LSTM (Bi-LSTM), and Gated Recurrent Unit (GRU), to detect malicious URLs with high accuracy rates of 97.0%, 99.0%, and 97.5% respectively, using datasets of both malicious and benign URLs. LSTM networks, belonging to the family of deep learning-based approaches exhibit promising outcomes in the detection of fake news. LSTM, a variant of Recurrent Neural Networks (RNNs), possesses the capability to capture long-term dependencies and patterns within textual data [15]. LSTMs are effective in detecting fake news due to their proficiency in learning from temporal dependencies within text data. Nevertheless, these models may face limitations in capturing both local and long-term relationships in the input.

The CNN+LSTM approach combines the strengths of both CNNs and LSTMs. The spatial properties of text data are well captured by CNNs, whereas the temporal relationships are well captured by LSTMs. By combining these two approaches, the CNN+LSTM approach can capture both the spatial and temporal features in text data, leading to improved accuracy in the identification of bogus news. The CNN+LSTM technique is successful in spotting fake news, according to several investigations. For instance, Umer et al. devised a hybrid deep learning model that synergizes CNNs and LSTMs for the purpose of detecting false news, yielding an impressive accuracy of 97.8% [18]. These findings serve as evidence for the efficacy of the CNN+LSTM approach in fake news identification.

To enhance the performance of false news detection models even further, recent advancements in deep learning have introduced skip connections [19]. Skip connections provide shortcuts between the neural network’s several layers, allowing the model to retain and propagate important information from early layers to later layers [20]. By leveraging skip connections, the model can capture both low-level and high-level features in the text, leading to improved accuracy in fake news detection. With skip connections, training deep networks becomes more feasible and efficient. They help to stabilize the training process, enabling the use of more layers in the network without sacrificing performance. Skip connections have shown promising outcomes across diverse computer vision and the problem of processing natural language, and their integration into fake news detection models holds great potential for enhanced performance. The integration of skip connections can reduce the manual efforts required for the identification and verification of news sources, improving the efficiency of news verification.

Now, traditional embedding methods generate word representations on the basis of other terms that appear within a text corpus. While they can capture some of the meaning of individual words, they may not capture the nuances of language that depend on the context in which they appear [21]. This can lead to inaccuracies in fake news detection [6]. Bidirectional Encoder Representation of Transformers (BERT) is a transformer-based language model that generates high-quality contextualized word embeddings, which can improve the performance of NLP tasks [22]. Contextual embedding models like BERT, take into account the entire context of a word when generating its representation. This allows them to capture the subtle relationships between words and their meanings, making them more effective for fake news detection [6, 23].

Trained language models, like BERT, have been successfully used to detect bogus news in the past, according to some studies [24–26]. For instance, BERT’s accuracy in classifying false news stories was 98.90% in research by Kaliyar et al. [24]. Similar to this BERT was employed in research by Fawaid et al. [25], to identify false news with an accuracy of up to 90%. With an accuracy of 92.4%, the AugFake-BERT model performs better than the twelve cutting-edge models [26], demonstrating the significance of a balanced dataset in classification performance.

By leveraging skip connections in combination with CNNs, LSTMs, and pre-trained contextual embeddings, our proposed model aims to develop a reliable and effective technique for detecting false news. The model we suggest builds upon this research by combining CNN and LSTM with pre-trained BERT embeddings that can detect both local and long-term relationships in given data, while also benefiting from the high-quality embeddings generated by BERT. The inclusion of skip connections allows our model to learn increasingly complex patterns, as in the relationships between words or phrases, as well as the presence of certain linguistic features indicative of fake news. Besides, we are using a deep CNN architecture. This can allow our model to learn increasingly complex textual patterns, like the relationships between words or phrases. By learning these high-level representations, the model can become more resistant to data noise as well as more accurately represent the text’s underlying structure. This research aims to enhance the precision and dependability of current false information detection models, which often struggle with identifying subtle forms of misinformation. We are confident that this proposed model will play a vital role in combating the dissemination of false news and misinformation across social media platforms. The overall contribution of this study includes:

- Our pioneering work, FakeStack, combines BERT, deep CNN with skip convolution block, and LSTM to introduce a novel approach that enhances the precision and robustness of identifying bogus news.
- Evaluated the individual contributions and interactions of skip connections, BERT, deep CNN, and LSTM within the hybrid model. The objective is to analyze how each component enhances the ability of the model to represent contextual data, detect semantic patterns as well as model sequential dependencies in news content.
- We reuse the features extracted from earlier layers in the network, promoting the efficient use of the learned representations. This leads to better generalization and faster convergence.
- We conducted extensive experiments to analyze the effect of various hyperparameters on how well the model performs, providing insights into the best practices for training deep learning models for detecting false news.
- Using a benchmark dataset, our suggested model reached state-of-the-art performance, proving the usefulness of the method. Our work contributes to the growing body of research on using deep learning techniques for combating the propagation of bogus news on social media.

The following is how this document is organized: The section Literature Review emphasizes relevant work in this sector; the Section Proposed Methodology demonstrates the suggested methodology; the Section Evaluation discusses the findings of our trials. In Section Limitations and Future Work, we discuss the study’s limits and suggested future studies. Finally, we draw the paper to a close in Section Conclusion.

## Literature review

Fake news is a growing problem in today’s information landscape. As the volume of online information continues to increase, distinguishing between truth and falsehood has become progressively challenging. Fake information can spread quickly through social media, with potentially harmful consequences for individuals, institutions, and society in general. Over the past few years, researchers have devised numerous machine-learning techniques to identify and combat false news.

One approach that has shown promise is the application of models based on deep learning. In that work, Yang et al. [27] introduced a CNN-based model for detecting bogus news. Their model takes the text of an article as input and uses multiple convolutional filters to extract features. The authors demonstrated that their CNN model outperformed several baseline models on a collection of actual and phony news stories. Kaliyar et al. [28] introduced a deep CNN model that extracts multiple features at each layer to detect false news. Their method was compared with several baseline models, as well as the proposed model demonstrated exceptional performance on benchmark datasets, surpassing existing approaches with a remarkable test-data accuracy of 98.36%. Nasir et al. [9] proposed a new hybrid deep learning model for detecting bogus news that incorporates convolutional as well as recurrent neural networks. The model underwent testing using two datasets specifically for false news analysis (ISO and FA-KES) and showed significantly better detection performance than approaches without hybridization or a combination of techniques used as baseline models. Moreover, the results of additional experiments on the model’s generalization across other datasets were promising. Poligraph presents an intrusion-tolerant and decentralized system for detecting fake news [29]. Central to Poligraph is a dual-layer consensus mechanism that effectively integrates both machine learning methods and human expert evaluation. This two-layer consensus framework is built using Byzantine fault-tolerant (BFT) and asynchronous threshold common coin protocols. The authors showcased Poligraph’s capabilities, achieving a throughput surpassing 5,000 transactions per second and demonstrating a remarkably low latency of 0.05 seconds. Sedik et al. [30] introduced a Deep Learning-based method for detecting fake news in the context of social media platforms. The proposed system utilizes GLOVE word representations for text encoding, followed by feature extraction and classification using various Deep Learning models, including CNNs. Experimental results show significant improvements in detection accuracy, particularly with the Concatenated CNNs (C-CNNs) algorithm, which achieved a remarkable accuracy rate of 99.6%, demonstrating the potential of CNN-based Deep Learning methods in addressing the issue of fake news on social media.

Over the past few years, scholars have also explored network-based approaches in the field of false information detection. Shu et al. [31] developed a new multimodal fusion network called the Fine-grained Multimodal Fusion Network (FMFN) that aims to identify false news by fully integrating textual and visual information. Their approach achieved comparable results to other fusion methods that combine visual and textual features, demonstrating that the FMFN’s joint representation, which integrates multiple visual and textual features outperforms representations obtained by fusing only visual or textual features in detecting fake news. Wang et al. [32] introduced the Curriculum-based Multi-Modal Masked Transformer Network (CMMTN), a novel approach to detecting fake news in the digital age. CMMTN addresses the challenge of limited labeled data by employing positive unlabeled (PU) learning and combines text and image information using advanced techniques like BERT and ResNet while also effectively masking irrelevant context between modalities. Experimental results on real datasets demonstrate its effectiveness in multi-modal fake news detection, making it a promising contribution to combating misinformation online.

Contextual embedding has become increasingly important in the domain of detecting bogus news, enabling the greater potential for a comprehensive understanding of the language used in news articles. Unlike traditional embeddings like GloVe, contextual embedding methods such as BERT take into account the surrounding words and phrases when generating word representations [22]. This is crucial in fake news detection since the surrounding context in which a term is utilized holds substantial influence over its interpretation and purpose.

Research has shown that contextual embedding models outperform traditional embedding models among tasks for identifying bogus news [5]. For instance, Jwa et al. [33] concentrate on utilizing data-driven methods for bogus news detection. BERT is applied to analyze the correlation between the heading and the body text, and it is pre-trained with supplementary news data. The deep contextualization provided by BERT proves to be highly effective for the task, surpassing the performance of older cutting-edge models. In their research, Gundapu et al. [34] presented an approach for scrutinizing the trustworthiness of COVID-19 outbreak-related material posted on Social media. They employed a combination of three transformer models, including ALBERT, BERT as well as XLNET, in an ensemble approach to identify false information. This model was assessed as part of the “COVID-19 Fake News Detection in English” shared task at ConstraintAI 2021, where on the test set, it received a f1-score of 98.55%. This performance placed their system fifth out of 160 participating teams.

Amid the challenges posed by the surge in fake news within the digital age, a hybrid fake news detection system is introduced by Essa et al. [35], leveraging a BERT-based approach combined with light gradient boosting machine(LightGBM). The proposed system outperforms various state-of-the-art methods across different datasets in detecting fake news using headlines or full content. Zhang et al. introduced a BERT-based domain adaptation neural network for multi-modal fake news detection (BDANN), an end-to-end model designed for detecting multimodal false news on microblogging networks [36]. Among the three modules, to extract text and image characteristics, the multi-modal feature extractor employs the BERT as well as VGG-19 models. In order to create an enhanced dataset with bogus data, Keya et al. devised a text augmentation approach using the BERT language model [26]. The suggested method resolves the minority class issue and conducts classification using the AugFake-BERT model, which is trained on an augmented dataset, for performing classification. Twelve distinct cutting-edge models are used the assess the suggested technique. With an accuracy of 92.4 percent, the suggested model performs better than the current models.

Kaliyar et al. introduced FakeBERT, a deep learning approach that combines parallel blocks of a single-layer deep CNN with BERT, as well as various kernel sizes and filters, for improved performance in fake news detection [24]. These classification outcomes demonstrate the superior performance of FakeBERT compared to existing models, achieving an accuracy of 98.90%. Guo et al. [37] proposed a novel fake news detection model based on a multiscale transformer, which can effectively capture semantic information in mixed languages. Experimental results on real-world data demonstrate that the proposed method outperforms common baseline models by 2%–10% in accuracy, showcasing its effectiveness in detecting fake news in mixed language scenarios. Praseed et al. [38] addressed the challenge of detecting fake news in resource-constrained languages, mainly focusing on Hindi. It highlights the limitations of existing techniques that primarily target English or require manual translation. The proposed approach utilizes an ensemble of pre-trained transformer models, including XLM-RoBERTa (Cross-lingual Language Model—RoBERTa), mBERT (Multilingual BERT), and ELECTRA (Efficiently Learning an Encoder that Classifies Token Replacements), all fine-tuned for the task of fake news detection in Hindi. By leveraging this ensemble, the study demonstrates improved efficiency in detecting fake news in Hindi, effectively addressing the limitations posed by individual transformer models and contributing to the broader goal of combating misinformation in regional languages.

In conclusion, the development of reliable models for detecting bogus news is of utmost importance. While deep learning approaches have shown promise, there is still much work to be done with the aim of enhancing the precision and resilience models of this type. One research gap in previous studies shows the limited exploration and utilization of contextual information. Fake news often relies on misleading or manipulated context, but traditional models may not effectively capture nuanced contextual cues. Another gap is the underutilization of skip connections in the model architecture, which can help propagate important information throughout the network. Addressing these gaps by incorporating contextual embedding techniques and utilizing skip connections can enhance the accuracy and resilience of models designed for false news identification and it is the main goal of this study.

## Proposed methodology

**Fig 1:**
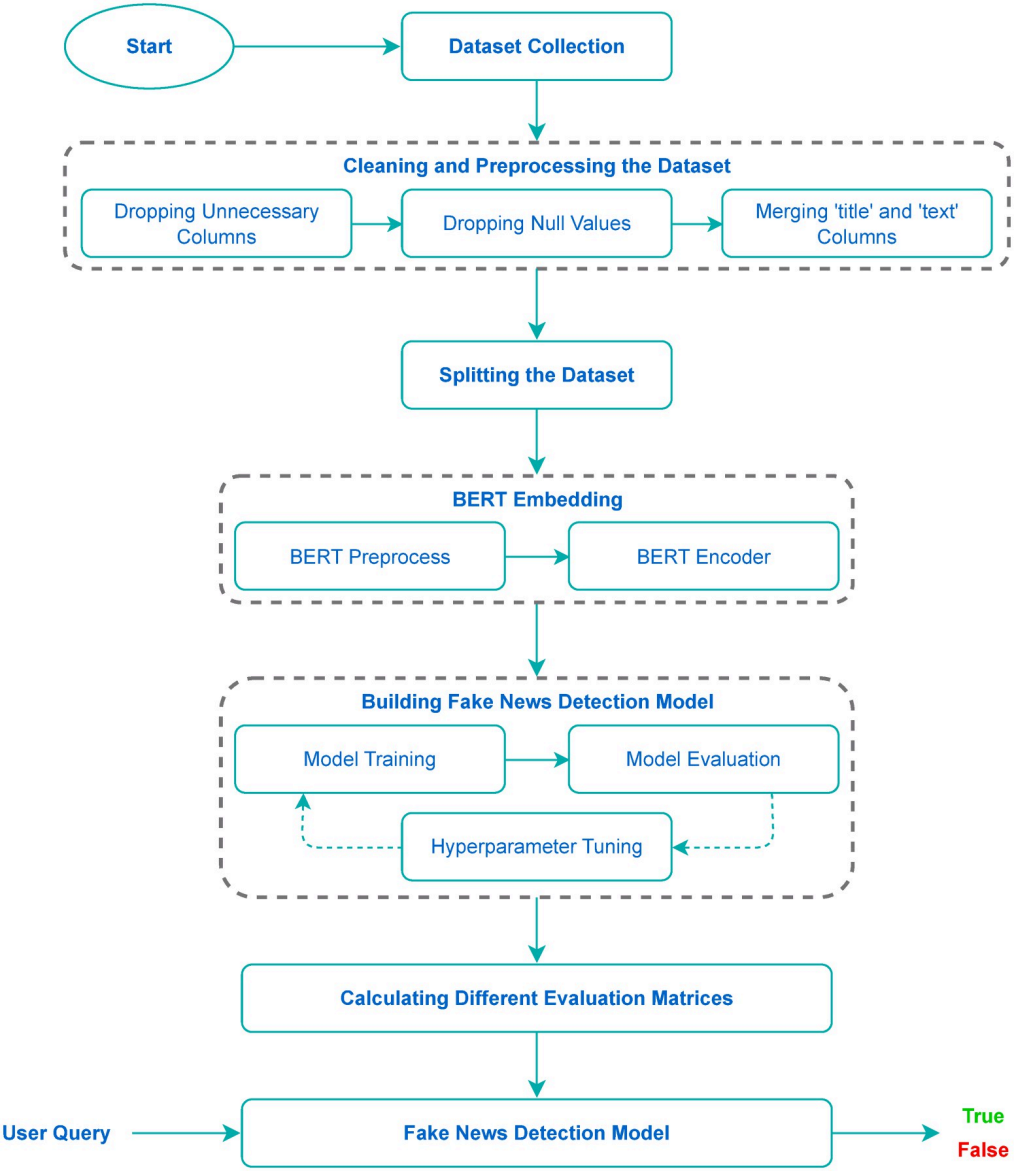
A flow chart of our proposed approach.

In today’s society, spreading false news presents a significant challenge, and its impact can be disastrous. As a result, it is crucial to develop effective techniques for bogus news detection. FakeStack utilized a deep CNN with skip connections and an LSTM model, with pre-trained BERT embedding. In order to provide a visual representation of the proposed methodology, we have constructed a comprehensive flowchart that illustrates the key stages of our approach. We can see this flow chart in Fig 1.

### Data collection

The initial stage in developing a false news detecting model was to collect and pre-process the dataset. The Fake News dataset utilized in this research was obtained from (https://www.kaggle.com/c/fake-news/data), which can be accessed by the public on Kaggle. Two CSV files make up the dataset, namely “train.csv” as well as “test.csv”. The file “train.csv” consists of 20,800 rows and 5 columns, representing news articles. Each row contains a unique identifier for the article, the article title, the author, the body text, and a binary label indicating whether the article is categorized as “fake” or “real” news. On the other hand, the “test.csv” file contains 5,200 rows and 4 columns, representing news articles as well. Each row contains a unique identifier for the article, the article title, the author, and the body text.

In an effort to fortify the validation of our proposed model, our analysis was extended to encompass two supplementary datasets, one of which is the LIAR dataset [6]. LIAR is a publicly accessible repository designed for the purpose of fake news detection. Comprising a decade’s worth of 9.9K manually labeled concise statements across diverse contexts, this dataset offers detailed analytical reports and source document links for each instance. The dataset comprises six columns, namely *News*_*Headline*, *Link*_*Of*_*News*, *Source*, *Stated*_*On*, *Date*, and *Label*.

The ultimate dataset, denoted as the WELFake dataset [47], comprises a total of 72,134 news articles, encompassing 35,028 instances of authentic news and 37,106 instances of fabricated news. To mitigate classifier overfitting and enhance machine learning training with a richer text dataset, the authors amalgamated four prominent news datasets (namely Kaggle, McIntire, Reuters, and BuzzFeed Political). The dataset is structured into four columns: *Serialnumber* (commencing from 0), *Title* (pertaining to the news heading), *Text* (reflecting the news content), and *Label* (where 0 denotes fake and 1 denotes real news).

### Data analysis and pre-processing

**Fig 2:**
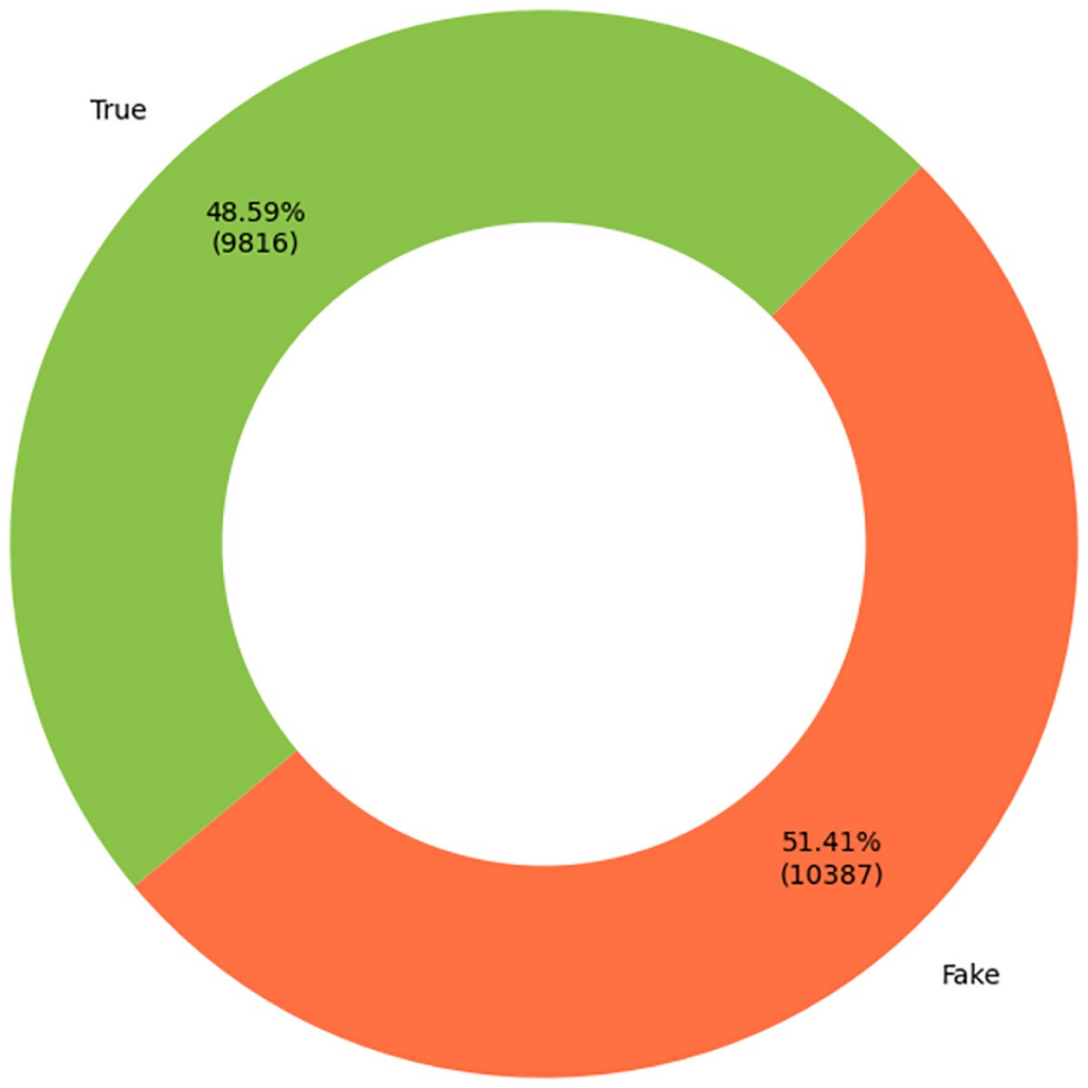
The amount of fake and true news.

In the initial stage of the study, the train.csv and test.csv files of the Fake News dataset were merged to form a unified dataset. The data was pre-processed by removing any null values and irrelevant columns that did not contribute to the analysis. The label column was encoded into binary values as it was initially in float. Subsequently, the title column, as well as the text column, were merged into a unified column. Some data samples are given in Table 1. After preprocessing the data, we analyzed the distribution of labels in the dataset and visualized it using the Seaborn and Matplotlib libraries. We found that the dataset exhibited a balanced distribution, and contains a similar amount of “fake news” as well as “real news”. We had 9816 True News and 10387 False News to work with as we can see from Fig 2. Finally, to ensure that the model generalizes well, using the *train*_*test*_*split* function, the data was divided into training and testing sets.

**Table 1:** Samples from the pre-processed dataset.

index	label	content
0	1	Ep. 544 FADE to BLACK Jimmy Church w/ Laura Eisenhower : Restoring the Balance [VIDEO] Click Here To ….
1	1	Poison By Dr. Mark Sircus Everyone knows that there are psychopaths everywhere including in the fields of medicine and .….
2	0	U.S. General: Islamic State Chemical Attack Had ‘No Impact’ on U.S. Forces WASHINGTON—U. S. and Australian troops ….
3	0	Breitbart News Daily: Trump Boom—Breitbart On the Thursday edition of Breitbart News Daily, broadcast .….
4	0	Avoiding Peanuts to Avoid an Allergy Is a Bad Strategy for Most—The New York Times This article originally ran in April. We are resurfacing it in light ….

### Embedding with pre-trained BERT

In recent years, natural language processing (NLP) has gained tremendous attention because of its uses in a variety of fields like sentiment analysis, language translation, and question-answering systems. One of the biggest breakthroughs in NLP is the BERT model’s development. BERT is a model that has been trained on a big corpus of text data [22]. This is a prominent method known for its proficiency in detecting and comprehending contextual significance from text [39]. During pre-training, a model is trained on an extensive dataset in an unsupervised manner to acquire generalized representations of the data. This pre-training process makes BERT a robust tool for a variety of NLP jobs, as the learned representations may be tailored for certain activities, like sentiment analysis as well as text classification.

BERT is fundamentally a stack of encoders within the transformer architecture. The transformer architecture comprises an encoder-decoder network employing self-attention on the encoder side and attention mechanisms on the decoder side. Notably, *BERT*_*BASE* features a stack of 12 layers within its encoder, whereas *BERT*_*LARGE* incorporates a more extensive stack with 24 encoder layers. To represent the text data in a machine-readable format, we used a pre-trained BERT embedding model. More specifically, we used version 3 of *bert*_*en*_*uncased*_*preprocess* for preprocessing. This module is available from TensorFlow Hub, and plays a vital role in preparing text data for integration with BERT models. Tailored for the “uncased” variant of BERT, where text is considered in lowercase, this module executes key preprocessing stages. It first tokenizes the input text, breaking it into constituent subwords or tokens. Special tokens like [CLS], denoting the sequence start, and [SEP], used for segment separation are then inserted. To ensure uniform input size, sequences are either padded or truncated. The module also assigns segment IDs to distinguish different segments within the input and generates attention masks to regulate token focus during processing. The tokenized and processed text is ultimately transformed into meaningful word embeddings, facilitating effective utilization within subsequent tasks. Then we used the “small_bert” model, specifically the version 2 of *bert*_*en*_*uncased*_*L* − 2_*H* − 128_*A* − 2 variant. This model is a reduced variant of BERT which has 2 layers in the encoder stack with a layer with a hidden size of 128 as well as another layer with two attention heads. This model has been trained on English text, with the input text lowercase (uncased) for generalization purposes. It can be customized for tasks like text categorization, sentiment analysis, and more. The *small*_*bert* models are designed to be computationally efficient while still providing reasonable performance for various NLP tasks. They are useful when we have limited computational resources or the model must be deployed on devices with limited resources.

In traditional NLP models, word embeddings are a method of representing a text’s words. as vectors in high dimensions. However, BERT uses a more sophisticated approach to word embedding. It leverages a pre-trained transformer-based model to generate word embeddings that capture contextual information. It captures the words’ meanings in the context of the entire sentence, rather than just the local context of the word. This makes the BERT embedding more powerful and effective for downstream NLP jobs, like text categorization.

### Convolutional Neural Network (CNN)

We begin by examining the CNN architecture. CNN model is well-suited for capturing local patterns and features in sequential data, making it effective for text classification tasks. They are mainly designed for image processing tasks but have also been applied in NLP jobs for their capacity to capture localized patterns in sequential data. CNNs possess the ability to automatically acquire hierarchical representations of the input data, which makes them very effective in extracting relevant features from text data. Researchers are attempting to enhance the efficacy of the bogus news detector by harnessing the capability of CNNs for feature extraction and classification [40].

A CNN model comprises a series of convolutional layers that convolve the input data using learnable filters, followed by non-linear activation functions, pooling operations for downsampling, and dense layers. The convolutional layers use filters of different sizes to derive useful characteristics from input embeddings. Max pooling reduces dimensionality and captures important features. Finally, dense layers are used for classification, utilizing the extracted characteristics. We incorporate dropout regularization to mitigate overfitting.

In contrast to a regular CNN, a deeper CNN has been shown to have a lower risk of overfitting [41]. Deep CNNs can automatically learn feature representations from the raw text, without the need for manual feature engineering. This means that the model can adapt to different types of text data as well as can be taught on extensive datasets without requiring human input. To further improve the classification process, Kaliyar et al. [28] introduced FNDNet, a deep CNN model with multiple hidden layers that effectively learns discriminative features for detecting false information. While this model exhibits reduced vulnerability to overfitting, it necessitates a longer duration for training. Overall, using a deep CNN can lead to better performance in fake news detection tasks, by enabling the system to acquire progressively intricate patterns within the text and by providing a more flexible and scalable approach to text classification.

#### Skip convolution block

Now, skip connections have emerged as a powerful technique for enhancing learning models’ effectiveness in various tasks. Within the realm of the identification of bogus news, skip connections have the potential to attain dependencies in textual data at both the local and global levels, leading to improved accuracy and robustness of the models.

By incorporating skip connections, CNNs can further enhance their ability to model complex relationships within news articles. Skip connections enable the direct propagation of information from early layers to deeper layers, enabling the flow of low as well as high-level characteristics.

The incorporation of skip connections in fake news detection models offers several benefits. Firstly, skip connections enable the models to capture both local and global dependencies, enabling a more holistic understanding of news articles. Secondly, skip connections facilitate the flow of information across different layers, helping the models preserve important features and gradients during training. Lastly, skip connections contribute to the overall interpretability of the models, as the skip connections explicitly reveal the significance of different layers in the course of decision-making. However, the utilization of skip connections in fake news detection models also presents certain challenges. One challenge is the increased complexity of the model architecture, which may require more computational resources for training and inference. Additionally, the optimal design and configuration of skip connections in the perspective of identifying false news is still an open research question, and further investigation is needed to identify the most effective strategies.

**Fig 3:**
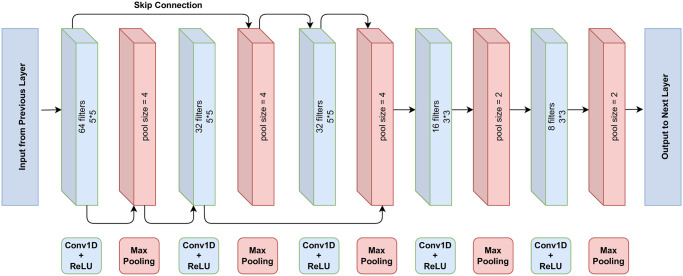
The deep CNN architecture used in our proposed model.

Within the framework of FakeStack, a sophisticated deep CNN architecture has been harnessed, as illustrated in Fig 3. Commencing with a dropout layer, the subsequent integration involves a 1D convolutional layer, implemented using the Keras “Conv1D” functionality, configured with 64 filters, a filter size of 5, and a ReLU activation function. This layer undertakes the extraction of local features from the input sequence through the employment of a sliding window mechanism. Subsequent to this, a pivotal dimensionality reduction is achieved via the inclusion of a max-pooling layer, orchestrated using the Keras “MaxPooling1D” module, featuring a pool size of 4. This pooling operation selectively retains the maximum value within each defined window, thereby enhancing computational efficiency. The architectural configuration proceeds with the addition of two further convolutional layers, each accompanied by an accompanying max-pooling layer. The convolutional layers are characterized by descending filter sizes of 32, each with a filter size of 5. Continuing this iterative pattern, the network deploys an additional set of convolutional layers, which further accentuates feature extraction. With filter sizes sequentially reduced to 16 and 8, and a window size of 3 for each layer, these convolutional layers are again accompanied by corresponding max-pooling layers with a pool size of 2. These layers extract more abstract features from the input sequence by gradually decreasing the spatial size of the feature maps.

The model architecture is ingeniously designed to harness the power of skip connections, strategically interwoven within the CNN layers. As the input data journeys through the network, it undergoes a sequence of convolutional transformations that unveil intricate features at different scales. Within this journey, the first notable instance of the skip connection arises after a convolutional layer, where its output is channeled directly into a subsequent layer. This ingenious arrangement, akin to a bridge between layers, dynamically fuses the extracted features with those from a higher-level convolutional layer. Moreover, another instance of the skip connection emerges, this time after a pooling operation. The result of this pooling layer is augmented with features from the previous convolutional layer, creating a symbiotic interplay between the finer-grained features and broader structural information. Collectively, these skip connections act as conduits for information transfer, amalgamating localized and global features. By adeptly integrating these skip connections, the model facilitates the seamless propagation of both detailed and holistic insights, culminating in a final layer poised to make informed predictions.

### Long Short-Term Memory (LSTM)

We look at a second architecture called the LSTM model. LSTMs belong to the family of recurrent neural networks (RNNs) and are specifically designed to capture long-term dependencies in sequential data [42]. By adopting a gating method to control the information flow, LSTMs, in contrast to conventional RNNs, overcome the vanishing gradient issue and may thereby detect long-range relationships in sequential data.

The cell state is an essential part of an LSTM, which acts as a memory unit that can capture and retain information over long sequences. Three major gates: the input gate, the forget gate, and the output gate, are used in a sequence of processes that update the state of the cell. Such gates, which are parameterized by learnable weights, allow the LSTM to selectively read, write, and forget information at each time step. The input gate controls the amount of new information that is added to the cell state, whilst the forget gate controls what information is discarded from the cell state. The output gate determines the amount of information that is exposed to the next layer or used for making predictions. By adaptively adjusting these gates based on the input sequence, LSTMs can effectively capture long-term dependencies and learn meaningful representations.

LSTM models have emerged as one of the leading approaches to solving various NLP problems. Their ability to model sequential data such as text has been demonstrated to be very effective. LSTMs have shown remarkable performance in tasks such as language modeling, machine translation, named entity recognition, etc. As it relates to our work, LSTMs hold promise for capturing the temporal dynamics and context within the news articles. By considering the word order and understanding the sequential nature of the text, LSTMs can learn representations that capture the meaning and semantics of the input, enabling accurate classification.

We incorporate LSTM layers into our model architecture to leverage their capability to capture long-term dependencies and extract informative features from the pre-trained BERT embeddings. By incorporating LSTM models into our methodology, we aim to leverage the contextual and spatial dynamics captured by LSTMs, thereby enhancing the discriminative capability of our model.

We introduce our innovative hybrid model for detecting bogus news in the next subsection that incorporates skip connections in a deep CNN and LSTM hybrid architecture, leveraging the benefits of both approaches to improve the detecting system’s resilience and accuracy.

### FakeStack

In our model, we incorporate pre-trained BERT embeddings. BERT, a cutting-edge transformer-based language model, is known for its rich contextual representations of words by considering their surrounding context. By leveraging the semantic connections among words, we can enhance the model’s comprehension of the input text and derive additional benefits. In our implementation, the first component of our model utilizes pre-trained BERT embeddings, specifically the “bert_en_uncased_preprocess” and “bert_en_uncased_L-2_H-128_A-2” models available through the TensorFlow Hub. The BERT embedding process begins with the input text data being preprocessed through the “bert_preprocess (bert_en_uncased_preprocess)” layer. This preprocessing layer serves to tokenize and encode the input text, generating enriched contextualized embeddings that encapsulate the semantic nuances of the text.

The preprocessed text is then routed through the “bert_encoder (bert_en_uncased_L-2_H-128_A-2)” layer, which further refines the embeddings by leveraging the power of BERT’s contextual understanding. This encoder layer employs a pre-trained BERT model, to perform contextual encoding. As a result, the output from the “bert_encoder” layer is an array of embeddings with enhanced contextual information, offering a comprehensive representation of the input text’s meaning. Subsequently, the sequence output obtained from the BERT encoder seamlessly transitions into the CNN architecture.

To capture local patterns and features within the text, we incorporate deep CNN layers into our model architecture. Multiple convolutional layers with varying filter sizes are utilized to capture different levels of granularity. By incorporating the Rectified Linear Unit (ReLU) activation function, the model’s capacity to capture intricate patterns is strengthened through the introduction of nonlinearity. Max pooling is applied to downsample the output feature maps and retain the most salient information.

We use skip convolution block in the deep CNN architecture to improve information flow even more and help the model train more successfully. Skip convolution blocks, also known as residual connections, allow the direct propagation of information from earlier layers to deeper layers. Specifically, skip connections are introduced between certain convolutional layers, allowing the direct flow of information from earlier layers to later layers. By introducing this, we facilitate the flow of both low-level and high-level features, enabling the model to better capture intricate patterns and dependencies within the text data. This helps mitigate the vanishing gradient problem and promotes better gradient flow during training, leading to improved convergence and overall model performance.

To capture long-term dependencies and sequential patterns, we employ LSTM layers in our model architecture. The LSTM layers get the output from the CNN layers as an input., creating a hierarchical architecture that first extracts local features using CNNs and then captures long-term dependencies using LSTMs. The LSTM layers utilize memory cells and various gating mechanisms to effectively capture the temporal dynamics and contextual information present in the input data.

CNNs excel at extracting local features and identifying patterns within short windows of the input data. This ability is particularly useful for capturing important local cues and linguistic structures in the text. On the other hand, LSTMs are adept at modeling sequential dependencies and capturing long-term relationships between these local features. By combining these two models, the input data can be learned as a hierarchical representation, with CNNs extracting local characteristics and LSTMs capturing the interactions and dependencies between these features over longer sequences.

**Fig 4:**
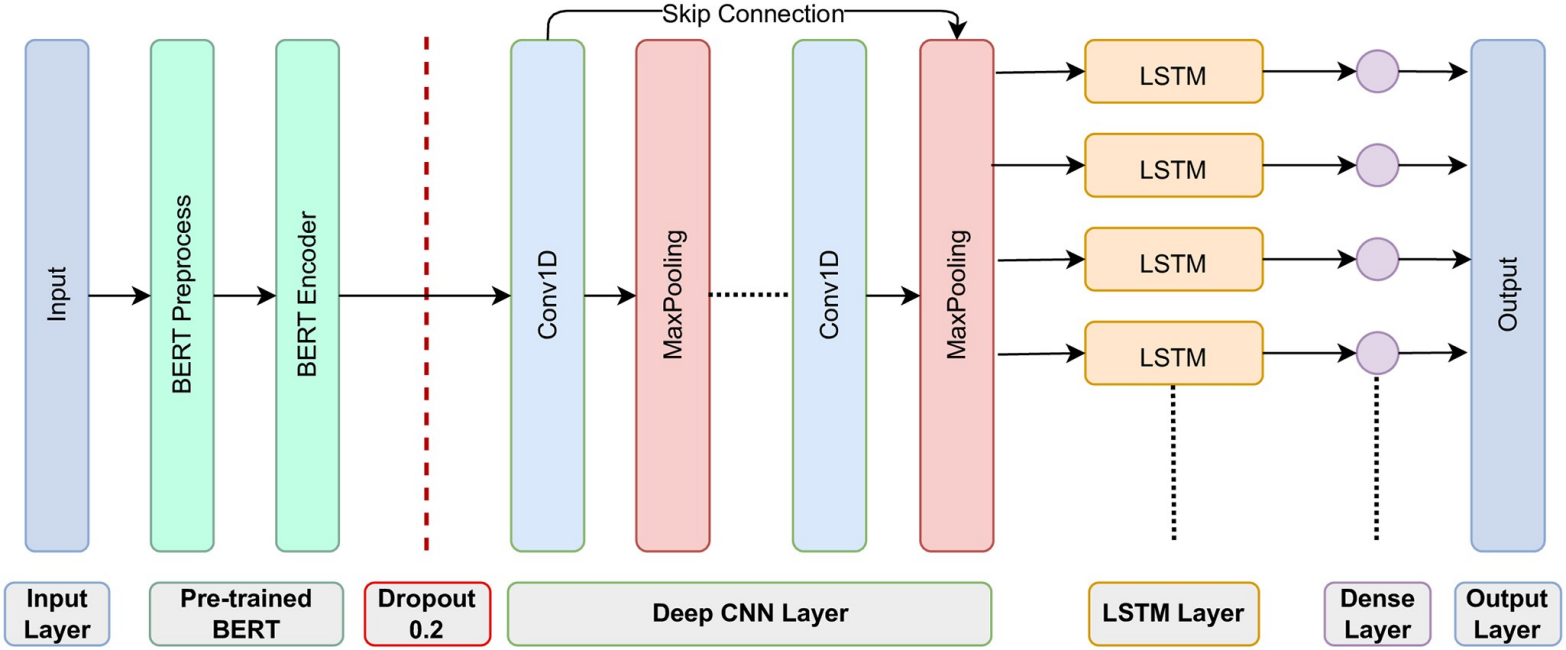
The proposed architecture.

We have devised a sophisticated stacked architecture, as illustrated in Fig 4, to enhance the precision and reliability of fake news detection. The architecture unfolds as follows: The initial input layer processes preprocessed data, followed by a pre-trained BERT layer that leverages BERT preprocess and encoder capabilities. To counter overfitting, a dropout layer of 0.2 is introduced before progressing to deep CNN layers for unveiling local features. The detailed view of our deep CNN layer is presented in Fig 3. Subsequently, outputs from these layers feed into the LSTM layer, capturing temporal nuances. The architecture concludes with a dense layer and an output layer, fine-tuning features and generating final classification outcomes. This whole process capitalizes on the strengths of both deep CNN and LSTM models, resulting in an effective and powerful model for false news identification and other NLP applications. By combining the unique capabilities of these two architectures, our design can capture local patterns and long-term relationships in text data simultaneously, leading to enhanced performance and improved generalization ability.

The integration of BERT embedding layers in our architecture allows us to effectively learn relevant features from the textual data. A unified feature is fed to the BERT model, which is a result of merging the title column and the text column. We named it as content feature. BERT’s pre-trained representations provide a strong foundation for understanding the semantics and context of the input text, which is crucial for accurate false news identification. We feed the extracted features with BERT through the CNN to learn more discriminative and hierarchical features. The deep CNN with skip block further enhances the model’s representational capacity. Skip connections enable gradient flow and facilitate the learning process in deep networks, mitigating the vanishing gradient problem. This not only allows for the efficient training of the model but also promotes feature reuse and information flow between different layers, resulting in better generalization. Furthermore, we incorporate the LSTM component into the stacked architecture. The learned features from the CNN are fed into the LSTM, which excels at modeling long-term dependencies and sequential patterns in the text data. LSTM’s ability to capture temporal relationships and dependencies is particularly valuable in understanding the context and contextually related information in news articles, further enhancing the model’s comprehension capabilities.

By leveraging the combined capabilities of these three components, our stacked model can effectively learn hierarchical representations of the input data. It comprehensively captures the nuances and semantic relationships between words, enabling a more nuanced understanding of the text and improving the model’s performance in various NLP tasks. Our proposed architecture outperforms standalone CNN or LSTM models in terms of accuracy and generalization ability. The model’s ability to handle both short-term and long-term connections in the data makes it particularly well-suited for false news identification, where identifying subtle patterns and contextual cues is crucial for accurate classification. Algorithm 1 showcases the details of our proposed model. By combining the strengths of deep CNN, LSTM, and BERT embedding layers, the algorithm effectively learns representations that capture the hierarchical nature of the input data. This comprehensive representation learning allows our model to achieve state-of-the-art performance in false news identification and other NLP tasks. Overall, our stacked model provides a powerful solution for false news detection and demonstrates how the combination of different architectural components can significantly improve performance and generalization ability in various NLP applications.

**Algorithm 1** Fake News Detection using FakeStack with Pre-trained BERT Embedding.

**Input**: Raw news dataset.

**Output**: Binary classification (Real/Fake).

1: Read dataset *D*.

2: Drop unnecessary columns and null values from *D*.

3: Split *D* into training and testing sets.

4: Embed the text sequences using pre-trained BERT model *B* to obtain *d*-dimensional embeddings:

$$\begin{array}{c} Xi,j=B\left(wi,j\right),\;\text{where}\;wi,j\in R^{k} \end{array}$$ (1)

Here, $Xi,j\in R^{d}$ is the *j*-th word embedding in the *i*-th news article, $w_{i,j}\in R^{k}$ is the *i*-th news article’s *j*-th word represented as a one-hot encoded vector, and *B*(⋅) is the BERT model that maps each word to its corresponding *d*-dimensional embedding.

5: Stack 1D convolutional layers and LSTM layer to build the fake news detection model *M*:

$$\begin{array}{c} h_{1}=\text{Conv1D}\left(X;\theta_{1}\right),\;\text{where}\;h_{1}\in R^{m\times d_{1}} \end{array}$$ (2)

  Skip Convolution:

$$\begin{array}{c} h_{1}=h_{1}+X,\;\text{where}\;h_{1}\in R^{m\times d_{1}} \end{array}$$ (3)

$$\begin{array}{c} h_{2}=\text{LSTM}\left(h_{1};\theta_{2}\right),\;\text{where}\;h_{2}\in R^{m\times d_{2}} \end{array}$$ (4)

Here, $X\in R^{m\times n\times d}$ is the input matrix of *m* news articles, each containing *n* words with *d*-dimensional embeddings, Conv1D(⋅; *θ*_1_) is a stack of 1D convolutional layers with parameters *θ*_1_ that extract features from the input sequence, LSTM(⋅; *θ*_2_) is a LSTM layer with parameters *θ*_2_ that captures the temporal dependencies between the features, and *d*_1_ and *d*_2_ are the output dimensions of the convolutional and LSTM layers, respectively.

6: Make the LSTM’s output flat and give it to a dense layer with sigmoid activation to get the final binary classification.:

$$\begin{array}{c} h_{3}=\text{Flatten}\left(h_{2}\right),\;\text{where}\;h_{3}\in R^{m\times d_{3}} \end{array}$$ (5)

$$\begin{array}{c} y=\sigma \left(h_{3}W+b\right),\;\text{where}\;y\in R^{m\times 1} \end{array}$$ (6)

7: Define the loss function of binary cross-entropy:

$$\begin{array}{c} L\left(ytrue,ypred\right)=-\frac{1}{m}\sum_{i=1}^{m}(y_{true,i}\;\text{log}\left(y_{pred,i}\right)+\left(1-y_{true,i}\right)\;\text{log}\left(1-y_{pred,i}\right)) \end{array}$$ (7)

where ***y**true* is the true binary label vector, ***y**pred* is the predicted binary label vector, and *m* is the number of news articles.

8: Initialize the model parameters *θ*_1_, *θ*_2_, ***W***, and ***b***.

9: Train the model *M* with Adam optimizer and binary cross-entropy loss function:

$$\begin{array}{c} \theta^{*}=\text{arg}\;\underset{\theta_{1},\theta_{2},W,b}{\text{min}}\;L\left(ytrue,ypred\right) \end{array}$$ (8)

10: Evaluate how well the model *M* is doing and adjust the hyperparameters accordingly if necessary.

11: Make predictions on the test set utilizing the trained model *M* to obtain the binary labels:

$$\begin{array}{c} \hat{y}=\text{argmax}\left(y_{pred}\right) \end{array}$$ (9)

12: Output the predicted binary labels $\hat{y}$ as the final results.

### Evaluation

#### Evaluation metric

A critical stage in machine learning is assessing a model’s performance in predictive modeling. Even if a model shows a high classification accuracy during development, it is important to test its capability to address the given issue in different scenarios. In assessing the deep learning model’s performance, some performance metrics are used. Here we presented the most popular evaluation metrics. P stands for positive, T for true, F for false, and N for negative in the computation. So, TP denotes True Positive, FP denotes False Positive, FN is False Negative, and TN denotes True Negative. In the Eqs (10) to (14), their computations are as follows.

□ *ACCURACY*: The accuracy score, or classification accuracy, is a measure of the percentage that refers to the proportion of correct predictions created by a model from all of the forecasts. This can be calculated using the formula in Eq (10).

$$\begin{array}{c} Accuracy:\frac{TP+TN}{TP+TN+FP+FN} \end{array}$$ (10)

□ *PRECISION*: A classifier’s precision is a measure of its accuracy. It represents the proportion of true positive forecasts to overall positive predictions, which includes both true positives and false positives. The Precision can be calculated using the formula in Eq (11).

$$\begin{array}{c} Precision:\frac{TP}{TP+FP} \end{array}$$ (11)

□ *RECALL*: Recall, also known as sensitivity or true positive rate, measures the proportion of correctly identified positive data out of the total number of actual positive data that should have been identified. It can be computed using Eq (12).

$$\begin{array}{c} Recall:\frac{TP}{TP+FN} \end{array}$$ (12)

□ *F1-SCORE*: When the dataset is imbalanced, F1-score can be used to evaluate how well a model predicts each class accurately. This statistic is frequently applied in assessments of false news identification. Eq (13) is used to determine the F1-score.

$$\begin{array}{c} F1:2*\frac{Precession*Recall}{Precession+Recall} \end{array}$$ (13)

□ *LOSS*: Eq (14) is a mathematical formula that calculates the binary cross-entropy loss, which is also called the log loss. It is often used in problems where we have two choices to measure how close the guess is to the right answer. Here, *Loss*(*y*) represents the loss function used for binary classification. *y*_*i* represents the true label or target value, while ${\bar{y}}_{i}$ represents the predicted probability for the corresponding sample.

$$\begin{array}{c} Loss\left(y\right):y_{i}log\left({\bar{y}}_{i}\right)+\left(1-y_{i}\right)+log\left(1-{\bar{y}}_{i}\right) \end{array}$$ (14)

□ *ROC CURVE AND AUC*: The Receiver Operating Characteristic (ROC) curve shows how well a classification model is doing. It is a graph that helps us understand the performance of a model across various classification thresholds, showing its success in distinguishing between correctly identified positive cases and incorrectly identified positive cases. Plotting the True Positive Rate (Recall) against the False Positive Rate (FPR) metrics yields the curve. AUC stands for “Area Under the ROC Curve”, and captures the overall area under the ROC curve. Using Eq (15), the FPR can be calculated.

$$\begin{array}{c} FPR:\frac{FP}{FP+TN} \end{array}$$ (15)

### Experimental setup

We constructed a model based on deep learning using TensorFlow that can decide if news articles are “true” or “false”. Our model architecture, as depicted in Fig 4, is a combination of BERT layers and neural network layers. The neural network layers comprise Conv1D, MaxPooling1D, skip convolution block, LSTM, and Dense layers. This hybrid approach allows us to leverage the powerful contextual embeddings provided by BERT and the sequential modeling capabilities of neural networks.

To develop and evaluate the model, we utilized Python programming language and leveraged popular libraries such as Pandas, NumPy, and Keras. These libraries provided a rich set of functions and tools for efficient data preprocessing, model construction, training, and evaluation. The development and experimentation were conducted on the Google Colab environment, a free cloud-based platform specifically designed for machine learning and deep learning projects. This platform offered computational resources and made the model development a faster process.

We preprocessed the dataset and kept two features. The first feature is a combined representation obtained by merging the title column and the text column, while the second feature is the label feature giving us a binary value. To make sure our model works well, we divided our dataset into two parts: one for training and one for testing. We did this using a function called *train*_*test*_*split* from Scikit-learn. The group used for training had 15, 152 samples and the group used for testing comprised 5, 051 samples. This division allowed us to train the model on a substantial amount of data while ensuring a robust evaluation of an independent dataset.

During the model training phase, we compiled the model with the Adam optimizer with the binary cross-entropy loss function. The aforementioned combination allowed the model to effectively optimize the parameters and minimize the classification error. To check how well our model is doing, we used different measures like accuracy, precision, as well as recall. These metrics provided valuable insights into the model’s ability to correctly classify whether a news piece is “fake” if not “real”.

Finally, after training our model, we evaluated its performance on the test set. The reported performance metrics in our results section showcase the effectiveness of our model in accurately classifying news articles. The binary accuracy metric shows how often the model’s predictions are correct overall. Precision and recall give information on how well the model can correctly find true positives and keep false positives and false negatives to a minimum.

We experimented with several models like CNN, LSTM, BERT-CNN, and BERT-LSTM to compare with our proposed FackStack model. Table 2 showcases the hyper-parameters of these models while training. The first model, CNN, employs 1D convolutions, followed by max pooling and ReLU activations. It does not incorporate any recurrent layers or dropouts. The second model, BERT-CNN, combines pre-trained BERT, with 1D convolutions, max pooling, and ReLU activations. The dropout rate is set at 0.2 in this model to prevent overfitting.

**Table 2:** Hyper-parameters used in each model.

Model	Convolutions	Pooling	Activations	Recurrent Layers	Dropout Rate
**CNN**	Conv1D	Max	ReLU	-	-
**BERT-CNN**	Conv1D	Max	ReLU	-	0.2
**BERT-LSTM**	-	-	Tanh, Sigmoid	LSTM	0.2
**FakeStack**	Conv1D	Max	ReLU, Tanh, Sigmoid	LSTM	0.2
	Conv1D	Max	ReLU, Tanh, Sigmoid	LSTM	0.2
	Conv1D	Max	ReLU, Tanh, Sigmoid	LSTM	0.2
	Conv1D	Max	ReLU, Tanh, Sigmoid	LSTM	0.2

The third model, BERT-LSTM, differs from the previous models by utilizing LSTM recurrent layers instead of convolutions and pooling. A dropout rate of 0.2 is applied. Finally, the FakeStack model consists of multiple stacked layers, each consisting of 1D convolutions, max pooling, ReLU activations, and LSTM recurrent layers. The model is regularized by applying a 0.2 dropout rate between each layer.

### Result analysis and comparision

In this part, we examine and compare how well our suggested model can detect fake news. We use a mix of pre-trained BERT embedding and extra neural network layers for this analysis. We have some ways to measure how well the model can tell the difference between real and fake news. These ways are accuracy, precision, recall, F1-score, and AUC (area under the ROC curve).

We begin by checking how well our model is working on a set of 20203 news articles. These test results are provided:

- Accuracy: 99.74%
- Precision: 99.67%
- Recall: 99.80%
- F1-score: 99.74%
- AUC: 1.0

The test accuracy of 99.74% indicates the overall percentage of instances that were classified correctly. Precision yields a value of 99.67%. The recall rate is 99. 80%. The F1-score, which balances precision and recall, is calculated as 99.74%. These metrics collectively demonstrate the model’s ability to accurately classify news items into categories for real and fraudulent news.

We also implemented some other base models to compare our proposed model. Table 3 has a list of them. On the “Fake News” dataset, the following table evaluates the performance of different models. It consists of several models, including Naive Bayes, SVM, Logistic Regression, KNN, CNN, BERT-CNN, BERT-LSTM, and the proposed model called FakeStack. All models share a common input, derived from the “content” and “label” columns. The “content” column is formed by merging the preprocessed “title” and “text” columns. The selection of embedding techniques for each model is evident from the accompanying table, providing a comprehensive overview of the distinct embedding methodologies employed. Each model’s accuracy in classifying fake news articles is recorded in the table.

**Table 3:** Comparison table of the implemented models.

Dataset	Model	Embedding	Accuracy
Fake News [43]	Naive Bayes	TfidfVectorizer	89.90%
	SVM	TfidfVectorizer	96.67%
	Logistic Regression	TfidfVectorizer	59.41%
	KNN	TfidfVectorizer	80.76%
	CNN	Keras Embedding Layer	92.16%
	BERT-CNN	BERT	99.37%
	BERT-LSTM	BERT	99.66%
	**Proposed Model (FakeStack)**	**BERT**	**99.74%**

The results reveal interesting insights. Comparing the models, we observe a wide range of accuracies. Naive Bayes achieves an accuracy of 89.90%, indicating its ability to make reasonably accurate predictions. SVM outperforms Naive Bayes, achieving an accuracy of 96.67%, demonstrating its effectiveness in classifying fake news articles. Logistic Regression, however, lags behind with an accuracy of 59.41%, suggesting limited performance in distinguishing between real and fake news.

KNN performs better than Logistic Regression, achieving an accuracy of 80.76%, indicating its capability to identify patterns in the dataset. CNN demonstrates further improvement with an accuracy of 92.16%, showing its effectiveness in capturing complex features and making accurate predictions.

The BERT-CNN and BERT-LSTM models achieve high accuracies of 99.37% and 99.66%, respectively. These models leverage pre-trained BERT embeddings combined with CNN and LSTM architectures, resulting in improved performance.

Among all the models evaluated, the proposed FakeStack model demonstrates superior performance, achieving an impressive accuracy of 99.74%. This model combines advanced techniques and architectures, likely including BERT, CNN, and LSTM, to effectively classify fake news articles.

**Fig 5:**
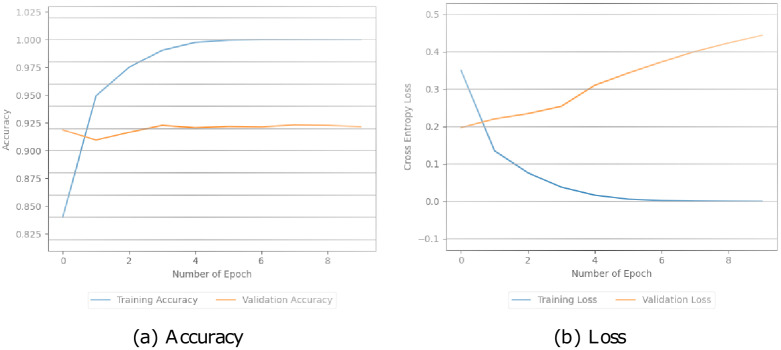
The accuracy and loss values of the CNN model.

**Fig 6:**
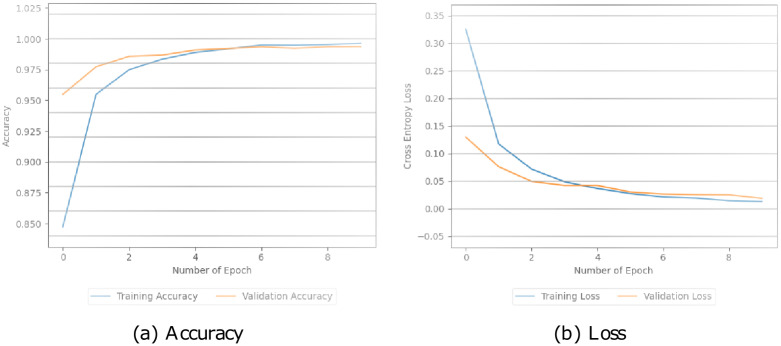
The accuracy and loss values of the BERT-CNN model.

**Fig 7:**
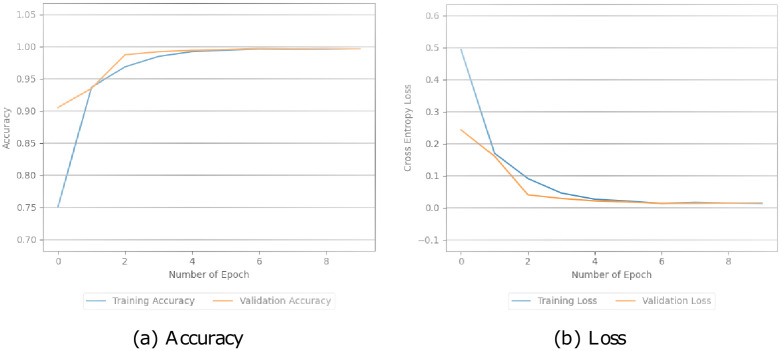
The accuracy and loss values of the BERT-LSTM model.

**Fig 8:**
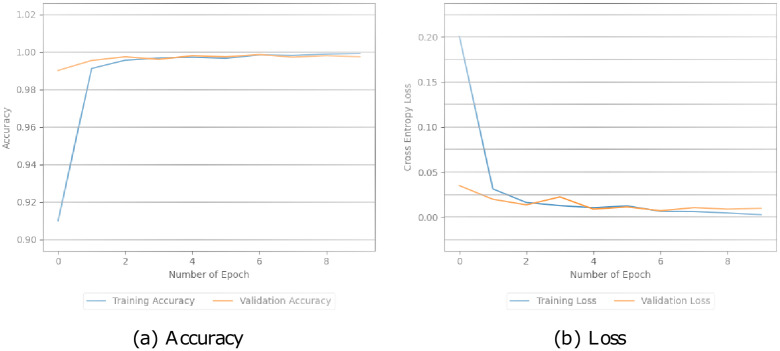
The accuracy and loss values of the FakeStack model.

Additionally, these models were assessed by looking at how well they performed during training and testing. This involved measuring their accuracy and loss during both stages in Figs 5–8. The simple CNN model achieved a perfect training accuracy of 100.00% but showed a significant drop in validation accuracy, indicating potential overfitting in Fig 5. The CNN model with pre-trained BERT exhibited high accuracy for both training (99.64%) and validation (99.37%), with relatively low training and validation losses in Fig 6. Similarly, in Fig 7, the BERT-LSTM model demonstrated comparable accuracy to the previous model, achieving 99.66% for both training and validation sets. Both models showcased effective learning and generalization with low losses. However, our proposed model performed well, with high accuracies (training: 99.87%, validation: 99.41%) and relatively low losses. This model stood out with the highest training accuracy among the models. We can see the values in Fig 8.

**Fig 9:**
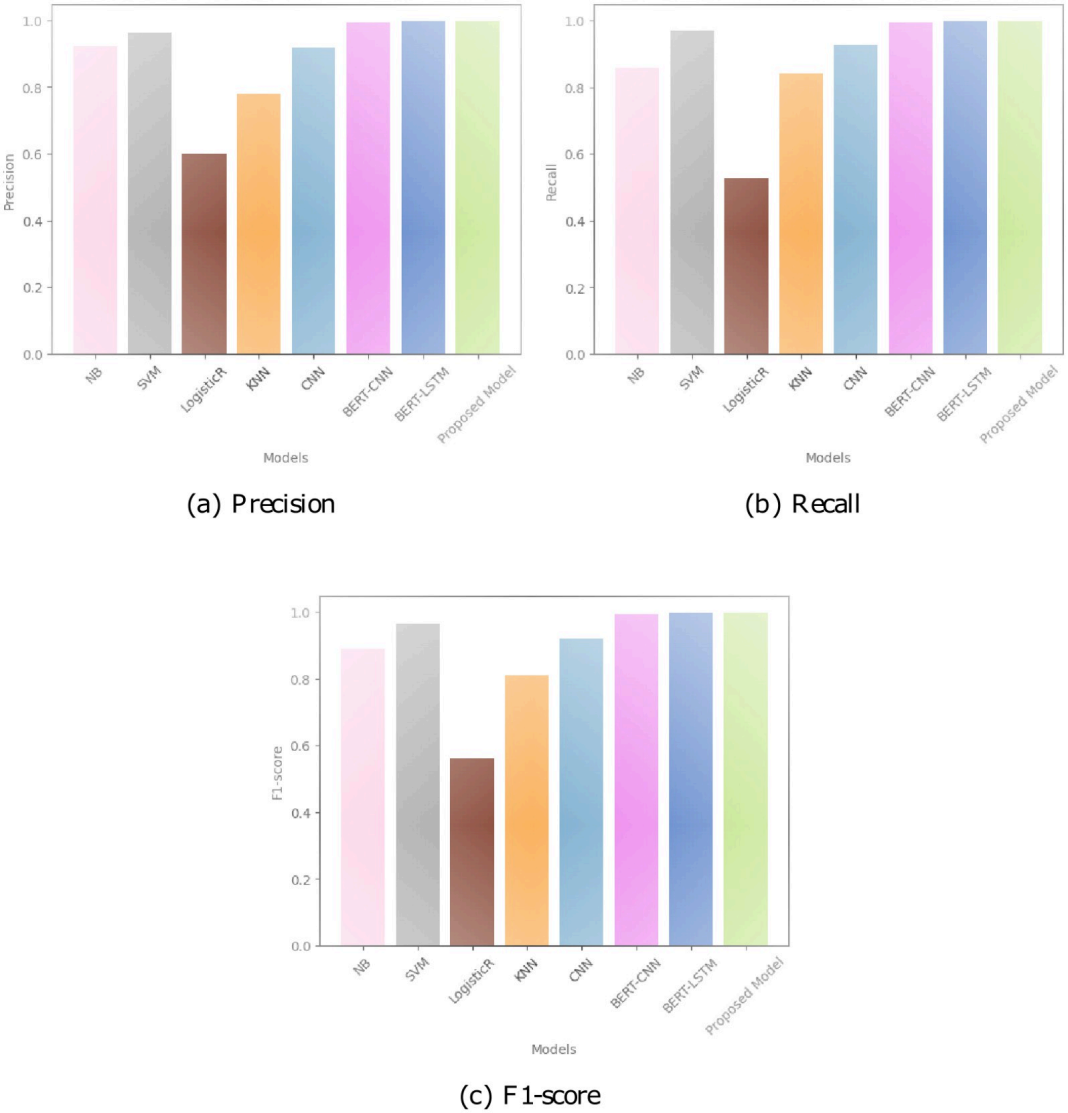
Comparing how well CNN, BERT-CNN, BERT-LSTM, and FakeStack models perform. A: Precision; B: Recall; C: F1-score.

The performance of the models, Naive Bayes, SVM, Logistic Regression, KNN, CNN, BERT-CNN, BERT-LSTM, and the Proposed Model, was assessed using Precision, Recall, and F1-Score metrics in Fig 9. Naive Bayes achieved a Precision of 0.9228, indicating its ability to correctly classify positive instances. However, its Recall of 0.8577 suggests that it struggled to capture all positive instances, resulting in some false negatives. The SVM model demonstrated high Precision (0.9613) and Recall (0.9708) values, signifying its effectiveness in accurately identifying both positive and negative instances.

Logistic Regression exhibited a Precision of 0.6006 and Recall of 0.5283, indicating its limitations in correctly predicting positive instances. This model displayed relatively lower performance compared to the other models. The KNN model showcased moderate Precision (0.9168) and Recall (0.9270) values, suggesting its ability to accurately classify positive instances while still leaving room for improvement.

The CNN model demonstrated substantial improvement with high Precision (0.9930), Recall (0.9938), and F1-Score (0.9934) values, indicating its strong performance in correctly classifying positive instances. BERT-CNN and BERT-LSTM models both exhibited outstanding Precision, Recall, and F1-Score values. The BERT-CNN model had a precision of 0. 9964, recall of 0. 9968, and F1-score of 0. 9966, highlighting its exceptional performance in accurately classifying positive instances. Similarly, BERT-LSTM demonstrated excellent Precision (0.9967), Recall (0.9980), and F1-Score (0.9974) values, showcasing its ability to correctly classify positive instances.

Among all the models, the Proposed Model stood out with the highest Precision (0.9967), Recall (0.9980), and F1-Score (0.9974) values. It exhibited superior performance in accurately classifying positive instances, surpassing all other models evaluated. The results highlight the potential of the Proposed Model, likely incorporating advanced techniques such as BERT embeddings, to effectively identify positive instances.

In short, when we look at the Precision, Recall, and F1-Score values, we can see that the different models perform differently. Among them, the proposed model demonstrated the highest performance, indicating its superior capability in accurately classifying positive instances. In all these models, we used the same hyperparameters which are enlisted in Table 4.

**Table 4:** Initial hyper-parameters.

Parameter	Parameter Value
Maximum Sequence Length	128
Epochs	10
Batch Size	32
Activation	ReLU, Sigmoid
Learning Rate	1 × 10^−3^
Optimizer	Adam

**Fig 10:**
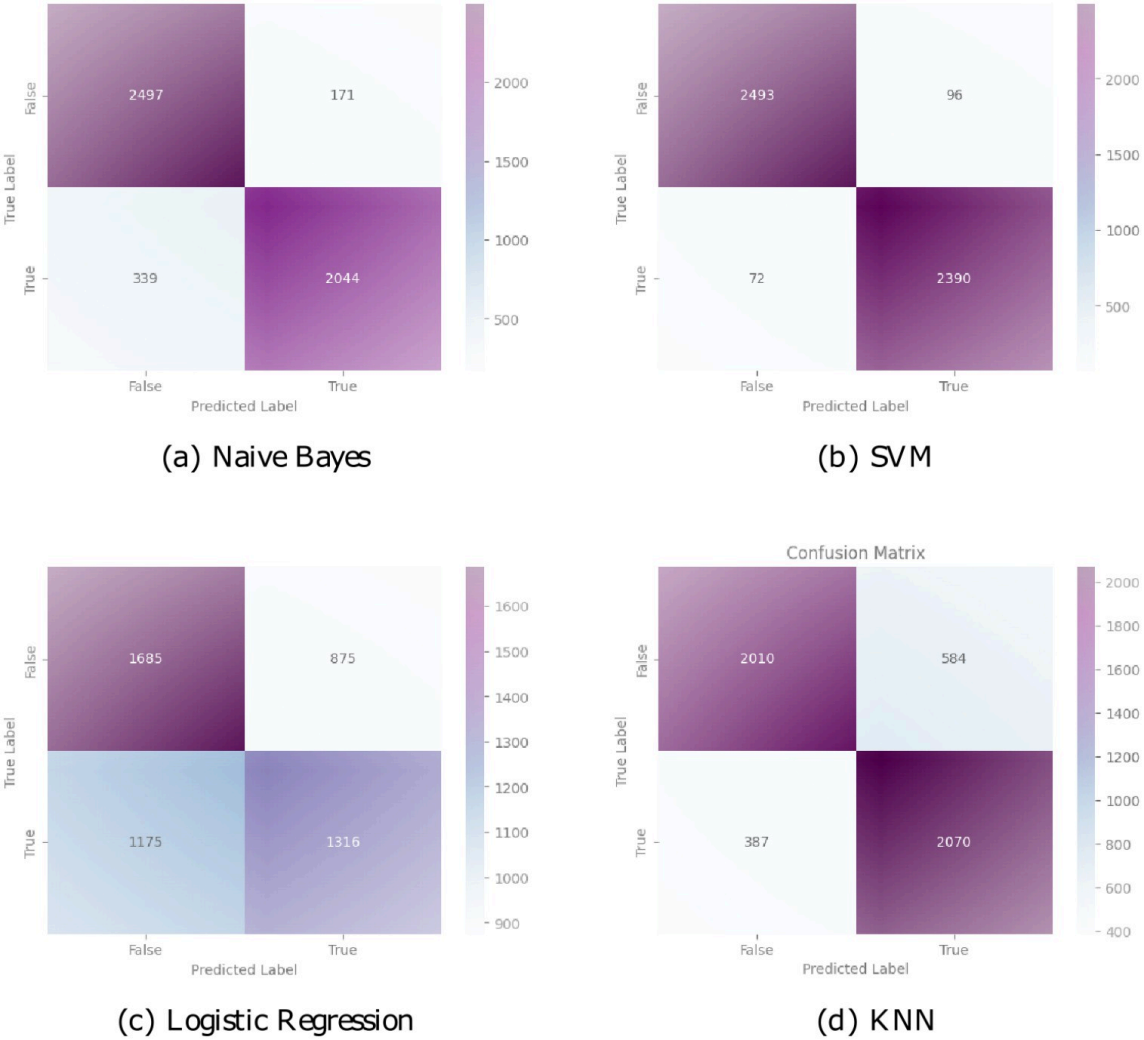
Confusion matrix for ML models.

**Fig 11:**
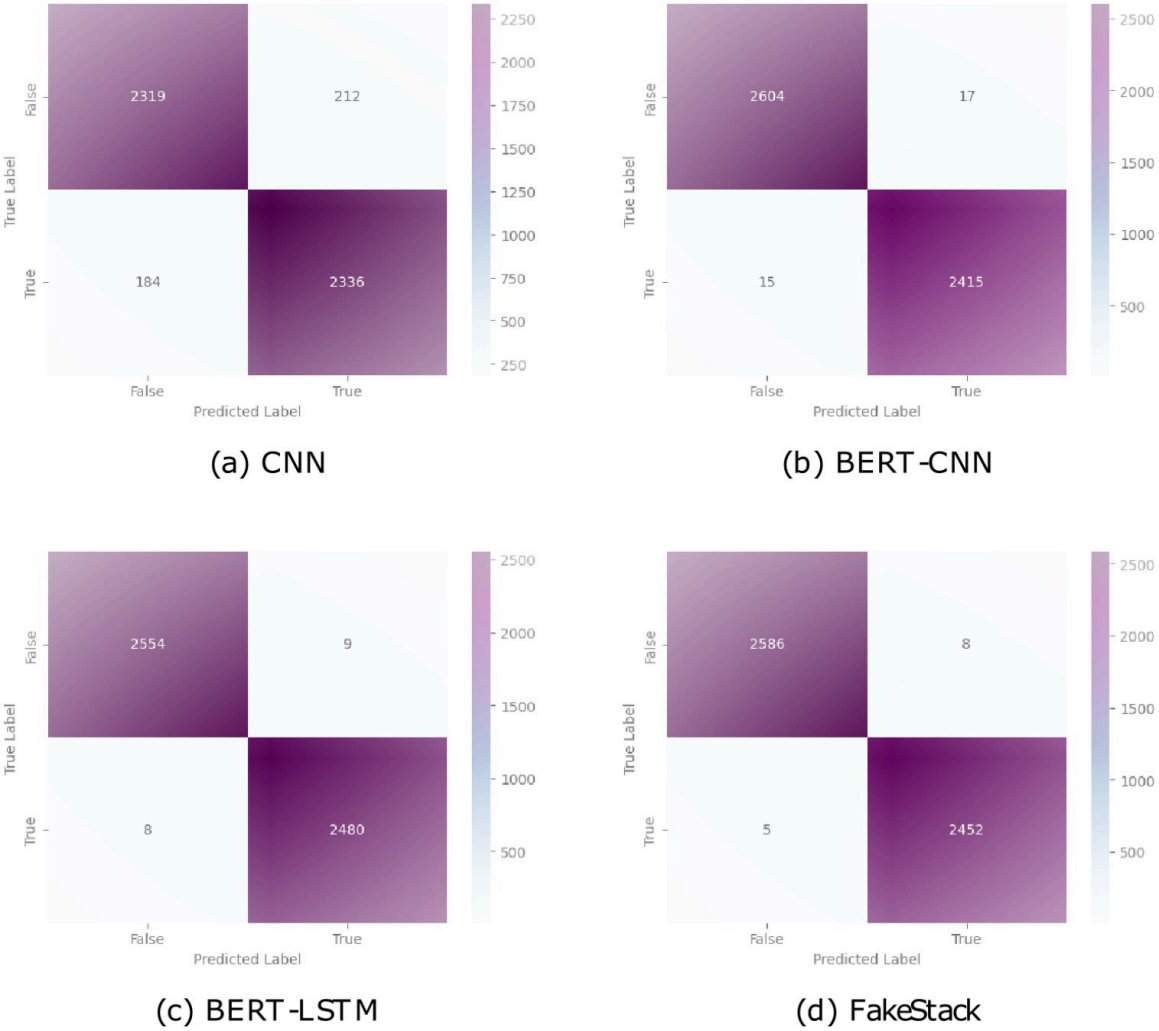
Confusion matrix for DL models.

To gain deeper insights into the model’s performance, we examine the confusion matrix, which displays the categorization outcomes. Figs 10 and 11 show the confusion matrix for our model as well as the other deployed models.

In Fig 10, for the Naive Bayes model, the confusion matrix revealed a true negative count of 2497, indicating the accurate classification of non-target instances, while there were 171 false positive cases where non-target instances were misclassified as targets. Additionally, the model exhibited 339 false negative cases and accurately classified 2044 true positive instances. In the case of the SVM model, the confusion matrix displayed 2493 true negative instances, illustrating the correct classification of non-target instances. However, there were 96 false positive cases, where non-target instances were incorrectly classified as targets. The model demonstrated a low false negative count of 72, suggesting its ability to accurately classify most target instances, along with a high true positive count of 2390. Logistic Regression exhibited a confusion matrix with 1685 true negative instances, demonstrating its effectiveness in correctly identifying non-target instances. However, the model had a high false positive count of 875, indicating the misclassification of non-target instances as targets. Moreover, Logistic Regression struggled with false negative cases, with 1175 instances misclassified, while correctly identifying 1316 true positive instances. The KNN model’s confusion matrix revealed a true negative count of 2010, demonstrating accurate classification of non-target instances. However, the model exhibited 584 false positive cases, where non-target instances were incorrectly classified as targets. Conversely, the model demonstrated 387 false negative cases, indicating instances that were misclassified as non-targets, along with a high true positive count of 2070.

The CNN model’s confusion matrix revealed a true negative count of 2319, indicating the accurate classification of genuine news articles. However, it exhibited a relatively higher false positive count of 212 and a false negative count of 184, implying a propensity for misclassifying some genuine news as fake and vice versa. On the other hand, the BERT-CNN model exhibited substantial improvement, with a significantly reduced false positive count of 17 and a false negative count of 15, contributing to a high true positive count of 2415. This model demonstrated improved accuracy in recognizing legitimate from fraudulent news stories, as evidenced by the true negative count of 2604. The BERT-LSTM model showcased even better performance, with a true negative count of 2554 and a remarkably low false positive count of 9, and a false negative count of 8. These results signify the model’s ability to accurately classify news articles. The proposed model continued to enhance the classification process, as evidenced by its true negative count of 2586, false positive count of 8, and false negative count of 5. It achieved a commendable true positive count of 2452, further reinforcing its effectiveness in identifying fake news. Collectively, the evaluation of the confusion matrices suggests that the BERT-CNN, BERT-LSTM, and the proposed model consistently outperformed the CNN model in differentiating between real and bogus news, with the recommended model exhibiting the highest level of accuracy and precision in Fig 11. These findings underscore the potential of advanced models, such as the proposed model, in combating the proliferation of bogus information and making sure that accurate information is shared.

**Fig 12:**
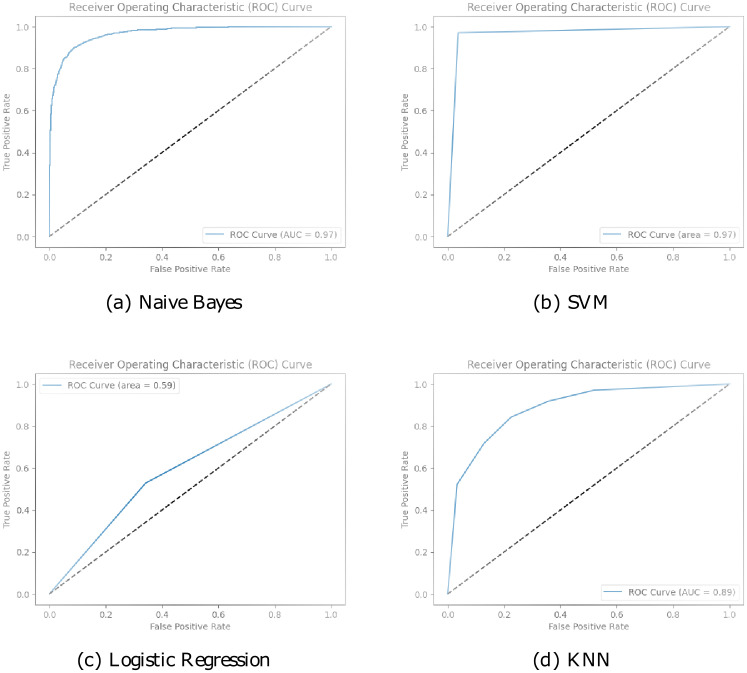
ROC curves for ML models.

Additionally, in Fig 12, the higher ROC curve values observed for Naive Bayes and SVM models indicate that they outperform Logistic Regression and KNN in terms of discriminatory power and prediction accuracy. Naive Bayes and SVM models demonstrate a remarkable ability to correctly classify instances, with an AUC-ROC score of 0.97. On the other hand, the Logistic Regression and KNN models, with an AUC-ROC score of 0.59, showcase a comparatively lower performance.

**Fig 13:**
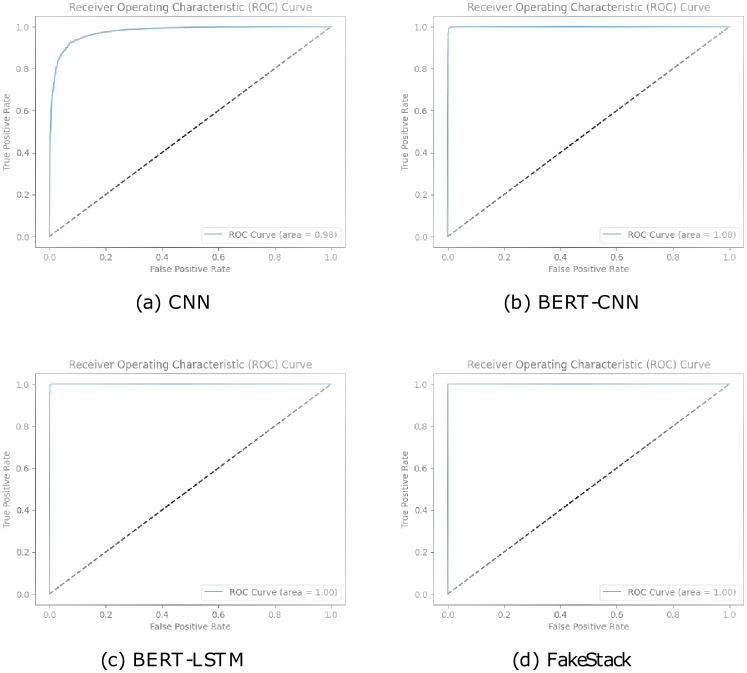
ROC curves for DL models.

The excellent ROC curve values obtained for BERT-CNN, BERT-LSTM, and FakeStack highlight their superior discriminatory power and predictive accuracy in identifying fake news in Fig 13. These models leverage the power of pre-trained language models like BERT, to capture nuanced semantic and contextual information, enabling them to make more informed decisions. The proposed FakeStack model, which incorporates novel architectural elements and advanced feature extraction techniques, further enhances the classification performance, achieving a perfect AUC-ROC score.

Although the CNN model achieved a slightly lower AUC-ROC score of 0.98, it still demonstrated impressive performance in distinguishing between fake and genuine news. The convolutional layers of the CNN model effectively capture local features and patterns, contributing to its strong discriminative ability. The results suggest that CNN-based models can also be valuable in fake news detection tasks.

In pursuit of deeper insights, we conducted additional experimentation using the implemented model, as highlighted in Table 5. This involved assessing the performance of the CNN, BERT-CNN, BERT-LSTM, and the Proposed Model, each exclusively employing the “title” column as input while maintaining consistent hyperparameters across the evaluations. The outcomes revealed an accuracy of 92.59% for the CNN model, 94.17% for the BERT-CNN model, and 93.23% for the BERT-LSTM model. Impressively, the Proposed Model continued to exhibit its superiority by achieving the highest accuracy of 94.49%.

**Table 5:** Comparison table of the implemented models with only “title” column.

Dataset	Model	Embedding	Accuracy
Fake News [43]	CNN	Keras Embedding Layer	92.59%
	BERT-CNN	BERT	94.17%
	BERT-LSTM	BERT	93.23%
	**Proposed Model (FakeStack)**	**BERT**	**94.49%**

To check if FakeStack works well, Table 6 compares FakeStack’s performance with existing methodologies for detecting false news. This table presents a performance comparison of existing models on the dataset “Fake News”. A number of models were evaluated according to their accuracy in classifying news articles as real or fake. Each model’s accuracy is listed in the table, along with a reference to the original source.

**Table 6:** Comparing the performance of different methodologies on the “Fake News” dataset.

Dataset	Model	Split	Accuracy
Fake News [43]	DT+LR+BGC [44]	-	88.08%
	LSTM+CNN [45] **Proposed Model (FakeStack)**	80-20	94.71% **99.70%**
	Merged CNNs [46] **Proposed Model (FakeStack)**	90-10	96% **99.60%**
	FNDNet [28]	-	98.36%

First, in the list, Sangamnerkar et al. [44] address the pressing issue of fake news, exploring ensemble techniques for binary news classification. It highlights source diversity to enhance accuracy and evaluates models using metrics like accuracy, precision, and recall. Without mentioning how they data partitioned their data, the top-performing approach combines Decision Tree, Logistic Regression, and Bagging Classifier with hard-voting for an accuracy of 88.08%.

On the other hand, by incorporating GloVe word embeddings, Agarwal et al. [45] merges convolutional and recurrent neural network architecture, achieving remarkable fake news prediction results. Tuned parameters and dropout layers enhance accuracy and precision (97.21%). However, employing the same splitting ratio as their model, our proposed approach outperformed their accuracy, achieving an impressive 99.70%.

Utilizing word embedding and convolutional neural networks, Amine et al. [46] integrate multiple metadata attributes for improved detection accuracy on real datasets. Employing a partitioning ratio of 90-10, their model attained a 96% accuracy, while our approach demonstrated superior performance with an accuracy of 99.60%.

Finally, FNDNet [28], a deep convolutional neural network for automatic fake news detection, overcoming the limitations of hand-crafted features. It achieves a remarkable accuracy of 98.36% on test data, without mentioning their splitting ratio. The research demonstrates the potential of CNN-based deep models in enhancing fake news detection across social media platforms.

In a bid to provide enhanced validation for our proposed model, we extended our analysis to encompass two additional datasets. One of these datasets, as depicted in Table 7, is the LIAR dataset. Initially we have 9960 instances that have been human-labeled. Subsequent to preprocessing, our dataset consisted of 6,180 instances classified as true and 2,378 instances classified as false. Keeping all the hyperparameters the same, the performance evaluation yielded accuracy scores of 73.95% for the BERT-CNN model and 73.07% for the BERT-LSTM model. However, our proposed model demonstrated superior performance, achieving an accuracy rate of 75.58%. The dataset exhibits a significant class imbalance, which has contributed to the observed lower accuracies.

**Table 7:** Comparison table of the implemented models with the second dataset.

Dataset	Model	Embedding	Accuracy
LIAR [6]	BERT-CNN	BERT	73.95%
	BERT-LSTM	BERT	73.07%
	**Proposed Model (FakeStack)**	**BERT**	**75.58%**

Lastly, our evaluation extended to the WELFake dataset, encompassing 72,134 news articles classified into 35,028 real and 37,106 fake news instances. Notably, this dataset comprises columns denoting *SerialNumber*, *Title*, *Text*, and *Label*. We effectively amalgamated the *Title* and *Text* columns, subsequently achieving accuracy metrics of 97.78% for the BERT-CNN model and 97.91% for the BERT-LSTM model. It is worth highlighting that our proposed model once again demonstrated its superior performance by achieving an accuracy rate of 98.25%. The overview of these findings can be found in Table 8.

**Table 8:** Comparison table of the implemented models with the third dataset.

Dataset	Model	Embedding	Accuracy
WELFake [47]	BERT-CNN	BERT	97.78%
	BERT-LSTM	BERT	97.91%
	**Proposed Model (FakeStack)**	**BERT**	**98.25%**

## Limitations and future work

The suggested model for identifying bogus news using a mix of BERT, deep CNN with skip connection, and LSTM has certain limitations. One limitation is its limited generalization capability. The model heavily relies on the availability and quality of labeled training data, and it may struggle to generalize to new or unseen sources of fake news that exhibit different patterns or characteristics. This highlights the need for diverse and representative training datasets to enhance the model’s ability to detect fake news across various sources.

Another limitation of the model is its computational complexity. The combination of BERT, deep CNN, LSTM, and skip connections can necessitate a significant amount of computer power and time to analyze, both for training as well as prediction. Efficient model architecture design and optimization techniques could be explored to mitigate these computational challenges and make the model better applicable to real-world situations.

Furthermore, the interpretability of the model poses a challenge. Deep learning models, including the proposed hybrid structure, are often considered black boxes due to their complex architectures. Interpreting the decision-making process and identifying the most influential features or factors for fake news detection may be challenging. Addressing this limitation by developing techniques such as attention mechanisms or visualization methods that highlight important words or regions within news articles would enhance the model’s interpretability and enable a better understanding of its predictions.

To address these limitations and further advance the field of fake news detection, several avenues for future work can be explored. Data augmentation techniques could be investigated to make the training data more varied and help the model for better generalization capability. In addition, transfer learning approaches using pre-trained models on larger datasets could be leveraged to enhance the model’s ability to detect fake news across different domains.

Incorporating multi-modal information is another promising direction. Fake news often involves various modalities, including text, images, videos, and social network dynamics. In the coming days, efforts could be made to fusion strategies and effectively integrate and analyze multi-modal data to enhance false news detection systems’ reliability and accuracy.

Furthermore, addressing the challenge of interpretability and explainability is crucial for building trust and understanding of the model’s decisions. Developing techniques to interpret and explain the model’s predictions, such as attention mechanisms or visualization methods that highlight important words or regions within news articles, would provide valuable insights into the factors contributing to the model’s classifications.

We can also try to improve the model to make it work better in real-time detection scenarios. Techniques like incremental learning or online learning could be explored to enable the model to adapt and update its knowledge in real-time without retraining from scratch, facilitating the detection of fake news as it emerges.

Finally, exploring the model’s vulnerability to adversarial attacks and developing techniques to enhance its robustness against such attacks is essential. Adversarial examples can be crafted to mislead the model into incorrect predictions, putting the accuracy of the system for detecting bogus news in danger. Investigating adversarial robustness and developing countermeasures would ensure the model’s effectiveness in real-world deployment.

Addressing these limitations and pursuing the suggested future work directions would contribute to the advancement of spotting bogus news., making them more accurate, efficient, interpretable, and robust in combating the spread of misinformation.

## Conclusion

Bogus information’s accurate detection is of paramount importance and ensures the integrity of information in today’s information-driven society. By leveraging the powerful contextual understanding provided by BERT embeddings, incorporating skip convolution blocks to propagate crucial information with harnessing the strength of deep CNN layers and LSTM, our proposed model FakeStack achieved remarkable accuracy and precision in distinguishing between real and fake news. The integration of these advanced techniques and methodologies not only enhances the model’s ability to combat the proliferation of fake news but also contributes to bridging the research gaps in leveraging contextual information, skip connections, and deep CNN architectures for improved fake news detection. The model’s performance was extensively evaluated with a baseline dataset “Fake News” using a comprehensive set of performance metrics, including accuracy, precision, recall, F1-score, and AUC. The results demonstrated that FakeStack outperformed other baseline models, showcasing its superior capability in accurately classifying positive instances. Furthermore, comparative analysis with existing approaches in fake news detection with different splitting revealed FakeStack’s exceptional performance, achieving an accuracy of 99.74%, which surpassed all previously reported accuracies. The high precision, recall, and F1-score metrics further validate the model’s effectiveness in correctly identifying fake news articles. FakeStack was also tested on the LIAR and WELFake datasets. Once more, in these two datasets, our model’s accuracy outperformed that of every other model. These findings highlight the potential of FakeStack and advanced deep learning techniques in combating the proliferation of fake news and ensuring the dissemination of reliable information in today’s information-driven society. Future research can explore the integration of additional features and data sources to further enhance the model’s performance and robustness.
